# Preliminary efficacy of pharmacological treatments on sluggish cognitive tempo (cognitive disengagement syndrome): a systematic review and meta-analysis

**DOI:** 10.3389/fpsyt.2026.1787612

**Published:** 2026-03-12

**Authors:** Şenay Kılınçel, Furkan Bulut, Pelin Göksel, Miraç Barış Usta, Oğuzhan Kılınçel

**Affiliations:** 1 Department of Child and Adolescent Psychiatry, School of Medicine, Istanbul Aydin University, Istanbul, Türkiye; 2 Private Practice, Psychotherapy Instute, Sakarya, Türkiye; 3 Department of Adult Psychiatry, School of Medicine, Ondokuz Mayis University, Samsun, Türkiye; 4 Department of Child and Adolescent Psychiatry, School of Medicine, Ondokuz Mayıs University, Samsun, Türkiye; 5 Department of Child Development, Istanbul Gelisim University, Istanbul, Türkiye

**Keywords:** cognitive disengagement syndrome, meta-analysis, pharmacological treatment, sluggish cognitive tempo, systematic review

## Abstract

**Objective:**

Sluggish cognitive tempo (SCT), also referred to as cognitive disengagement syndrome (CDS), is characterized by symptoms such as mental fogginess, slowed behavior, daydreaming, and reduced alertness. It is increasingly recognized as a construct distinct from attention-deficit/hyperactivity disorder (ADHD). This systematic review and meta-analysis aimed to evaluate the effectiveness of pharmacological interventions on SCT/CDS-related outcomes.

**Methods:**

A comprehensive literature search was conducted in PubMed, Scopus, and Web of Science. Studies assessing pharmacological treatments with reported SCT/CDS outcomes were included. The primary quantitative synthesis focused on randomized controlled trials and crossover designs, while open-label studies were analyzed qualitatively. Standardized mean differences (Hedges’ g) were calculated using a small-k-robust random-effects model (Paule–Mandel τ² estimator with Hartung–Knapp adjustment). A 95% prediction interval was additionally reported.

**Results:**

A total of seven studies were included in the qualitative synthesis, of which three (43%) provided sufficient data for meta-analysis. The total sample sizes of individual studies were generally small, contributing to limited statistical power. Using a small-k-robust random-effects model (Paule–Mandel τ² with Hartung–Knapp adjustment), the pooled effect was g=0.39 (95% CI: 0.01–0.78). The 95% prediction interval was −0.06 to 0.85. Between-study heterogeneity ranged from moderate to high (I² > 50%), reflecting variability in study design, pharmacological agents, outcome measures, and population characteristics.

**Discussion:**

Preliminary evidence suggests that pharmacological treatments, particularly atomoxetine and methylphenidate, may be associated with moderate improvements in SCT/CDS symptoms. However, given the limited number of controlled trials and the heterogeneity in populations (e.g., ADHD with comorbid dyslexia), these findings should be considered exploratory. Further large-scale RCTs are necessary to confirm these results and establish personalized treatment protocols.

## Introduction

Sluggish cognitive tempo (SCT), now increasingly referred to as cognitive disengagement syndrome (CDS), describes a constellation of attentional-behavioral symptoms characterized by excessive daydreaming, mental confusion/fogginess, lethargy, drowsiness, and slowed behavior/thinking (1, 2). In 2023, an international SCT Work Group reviewed the accumulated evidence and reached a consensus recommendation to adopt *CDS* as the preferred term, partly to better reflect the emerging science and improve acceptability for clinical and research use (3).

Over the past decade, the SCT/CDS literature has expanded rapidly, with substantial attention devoted to whether SCT/CDS represents a construct that is distinct from ADHD inattentive symptoms rather than merely another description of inattention (4–6). The most comprehensive quantitative synthesis to date is a meta-analysis by Becker and colleagues, which evaluated the internal, external, and diagnostic validity of SCT across the available literature through 2015. That meta-analysis found strong support for internal validity (factor-analytic differentiation from ADHD symptoms) and showed meaningful associations with multiple external correlates, while also highlighting major gaps—particularly around diagnostic classification and longitudinal prediction (7).

Parallel to construct-validation work, research has advanced on how SCT/CDS should be assessed. A systematic review focusing on SCT measurement over the past decade identified multiple SCT-specific instruments with generally acceptable-to-excellent reliability and structural validity, and it emphasized the importance of using validated SCT/CDS scales (rather than limited proxy items) when studying correlates and outcomes (8). This measurement foundation matters directly for treatment research: if SCT/CDS is assessed inconsistently across trials (different scales, informants, and symptom domains such as “daydreamy” vs “sluggish/sleepy”), true treatment effects may be obscured or appear inconsistent (5, 9).

Despite growing recognition that SCT/CDS is associated with clinically relevant impairment and psychosocial outcomes, evidence-based guidance on treatment remains limited. In particular, clinicians frequently encounter SCT/CDS symptoms in individuals evaluated or treated for ADHD, yet it is unclear whether standard ADHD pharmacotherapies reliably improve SCT/CDS symptoms, whether any improvement is independent of changes in ADHD symptoms, and whether certain SCT/CDS domains are more responsive than others (1, 10, 11). A recent “leading article” reviewing medication treatment of CDS identified only a small body of studies and concluded that the existing evidence suggests potential benefit from psychostimulants (e.g., methylphenidate, lisdexamfetamine) and atomoxetine, while emphasizing the need for replication and more systematic evaluation (12).

Within the limited pharmacotherapy literature, the pattern of findings is suggestive but not definitive. For atomoxetine, *post hoc* analyses of placebo-controlled data indicate that improvements in SCT can be partially independent of improvements in ADHD inattentive symptoms, supporting the possibility that SCT/CDS treatment response is not simply a byproduct of reduced ADHD inattention (13). For stimulants, an open-label trial in children treated with methylphenidate reported improvements in SCT total scores and certain SCT subdomains, while also noting complex relationships between baseline SCT dimensions and ADHD treatment response in home and school contexts (14). In adults with ADHD and comorbid SCT, a placebo-controlled crossover trial reported moderately large effects of lisdexamfetamine versus placebo on SCT ratings, though interpretation is complicated by carryover effects across treatment periods (15–17). Beyond trials, case-level clinical reports also reflect uncertainty and heterogeneity, for example, a published case report described greater improvement in SCT symptoms after switching from methylphenidate to atomoxetine, underscoring how individualized responses may vary and how sparse the evidence base remains (18).

Taken together, the field currently has a solid and growing foundation supporting SCT/CDS as a meaningful construct with established measurement tools, and a small but increasingly diverse pharmacotherapy literature suggesting possible benefit of several medications (19, 20). However, there is no dedicated meta-analysis that quantitatively synthesizes pharmacologic treatment effects on SCT/CDS outcomes across available studies, compares effects across medication classes, and evaluates moderators such as age group, study design (randomized vs open-label), informant (self/parent/teacher), and SCT/CDS symptom domains. This gap is increasingly consequential given the 2023 consensus shift toward CDS terminology and the rising likelihood that future clinical and research work will expect evidence summaries aligned to CDS/SCT outcomes rather than ADHD outcomes alone.

Therefore, the present study aimed to conduct a systematic review and meta-analysis of pharmacological interventions reporting SCT/CDS outcomes, synthesizing effect sizes for change in SCT/CDS symptoms and, where possible, examining whether SCT/CDS improvements track with or diverge from changes in ADHD symptoms. By integrating this fragmented literature quantitatively, we seek to clarify the magnitude and consistency of medication-associated changes in SCT/CDS and to identify methodological and clinical priorities for the next generation of trials.

## Materials and methods

### Study design and reporting standards

This systematic review and meta-analysis were conducted in accordance with the Preferred Reporting Items for Systematic Reviews and Meta-Analyses (PRISMA) 2020 guidelines (21). The study protocol was developed *a priori*, specifying eligibility criteria, outcomes of interest, and statistical methods, with the intention of prospective registration. All stages of study identification, screening, eligibility assessment, and inclusion were performed systematically and independently by two reviewers.

### Literature search strategy

A comprehensive literature search was conducted in PubMed/MEDLINE, Scopus, and Web of Science from database inception through December 2025. To ensure coverage of both historical and updated terminology, the search strategy combined terms related to sluggish cognitive tempo and cognitive disengagement syndrome with pharmacological intervention terms.

The core search string was (“sluggish cognitive tempo” OR “cognitive disengagement syndrome” OR SCT OR CDS) AND (pharmacotherapy OR pharmacological OR medication OR stimulant OR methylphenidate OR atomoxetine OR lisdexamfetamine). Search strategies were adapted for each database using controlled vocabulary where applicable (e.g., MeSH terms in PubMed). Reference lists of all included studies and relevant narrative reviews were manually screened to identify additional eligible publications. No restrictions were placed on publication status, but only peer-reviewed articles published in English were included.

### Eligibility criteria

Studies were considered eligible for inclusion if they fulfilled all of the following criteria: (i) the study population consisted of children, adolescents, or adults who were assessed for sluggish cognitive tempo (SCT)/cognitive disengagement syndrome (CDS) symptoms, either as a primary construct or as a distinct outcome within ADHD or related clinical populations; (ii) the intervention involved any pharmacological treatment, including stimulant medications (e.g., methylphenidate, lisdexamfetamine), non-stimulant agents (e.g., atomoxetine), or other centrally acting pharmacological agents; (iii) the study included a comparator condition, defined as placebo, no-treatment control, baseline assessment in pre–post designs, or an alternative pharmacological treatment; (iv) SCT/CDS outcomes were assessed quantitatively using validated or explicitly described rating scales; and (v) the study employed an eligible study design, including randomized controlled trials (parallel-group or crossover), open-label trials, or prospective observational studies reporting pre- and post-treatment SCT/CDS outcomes.

Studies were excluded if they met any of the following criteria: (i) SCT/CDS outcomes were not reported separately from general ADHD inattentive symptoms or could not be clearly distinguished as a distinct construct; (ii) the study focused exclusively on non-pharmacological interventions, such as behavioral, psychosocial, educational, or lifestyle-based treatments; (iii) the publication was a review article, meta-analysis, editorial, commentary, conference abstract, protocol, or case series without sufficient quantitative data to calculate effect sizes; (iv) the study involved animal models or *in vitro* experiments; (v) quantitative SCT/CDS data were not extractable, including absence of means, standard deviations, change scores, or other statistics required for effect size estimation; or (vi) the article was not published in English or was not available as a full-text peer-reviewed manuscript.

### Study selection process

All records identified through database searches were imported into reference management software, and duplicates were removed. Two reviewers independently screened titles and abstracts for relevance. Full-text articles were retrieved for potentially eligible studies and assessed independently against inclusion and exclusion criteria. Disagreements were resolved through discussion, and when necessary, by consultation with a third reviewer. The study selection process was documented using a PRISMA flow diagram.

### Data extraction

Data extraction was conducted independently by two reviewers using a standardized data extraction form. Extracted information included study characteristics (first author, publication year, country, and study design); sample characteristics (sample size, age range or mean age, sex distribution, and diagnostic status); pharmacological intervention details (type of medication, dosage, and treatment duration); SCT/CDS assessment methods, including the specific rating scales used and the type of informant (self-report, parent-report, or teacher-report); and quantitative outcome data, such as means, standard deviations, and change scores. When reported, ADHD symptom outcomes assessed concurrently with SCT/CDS were also extracted to allow exploratory comparisons. If outcome data were incomplete or presented only graphically, corresponding authors were contacted when feasible to obtain additional information. In studies reporting multiple SCT/CDS subscales or informant-specific scores, total SCT/CDS scores were prioritized for inclusion in the primary meta-analysis to ensure consistency across studies.

### Risk of bias assessment

Risk of bias was assessed independently by two reviewers. Randomized controlled trials were evaluated using the Cochrane Risk of Bias Tool version 2 (ROB 2) (2), assessing bias arising from the randomization process, deviations from intended interventions, missing outcome data, outcome measurement, and selective reporting (22). Non-randomized and observational studies were assessed using the Newcastle–Ottawa Scale (NOS) (23). Any discrepancies in risk-of-bias judgments were resolved by consensus.

### Effect size calculation

The primary effect measure was Hedges’ g, chosen to account for small-sample bias. For controlled studies, effect sizes were calculated as the standardized mean difference between intervention and comparator groups. For pre–post designs without a control group, within-group standardized mean change scores were calculated using reported means and standard deviations. When change-score standard deviations were unavailable, established imputation methods were applied using correlation coefficients derived from similar studies. Positive effect sizes indicated greater improvement (reduction) in SCT/CDS symptoms following pharmacological treatment.

### Statistical analysis

Given the small number of studies contributing to the quantitative synthesis (k=3), we re-estimated the pooled effect using a small-k-robust random-effects approach. Between-study variance (τ²) was estimated using the Paule–Mandel method, and 95% confidence intervals were computed with the Hartung–Knapp–Sidik–Jonkman adjustment. In addition to the pooled Hedges’ g, we report a 95% prediction interval to reflect the expected range of effects in a new study. Statistical heterogeneity was quantified using the I² statistic, with values of approximately 25%, 50%, and 75% interpreted as indicating low, moderate, and high heterogeneity, respectively. Where sufficient data were available, predefined subgroup analyses were performed according to medication class (stimulant vs non-stimulant), age group (children/adolescents vs adults), study design (randomized controlled trials vs open-label studies), and informant type (self-report vs parent- or teacher-report). Sensitivity analyses were conducted to evaluate the robustness of pooled effect estimates by excluding studies judged to be at high risk of bias and by sequentially omitting individual studies in a leave-one-out approach.

### Publication bias

While we initially planned to evaluate publication bias visually via funnel plots and statistically via Egger’s regression test, these methods require a minimum of 10 studies to provide sufficient statistical power. Because fewer than ten studies were available for quantitative synthesis in this preliminary meta-analysis, formal statistical testing for publication bias was not feasible and was therefore not performed.

## Results

**Figure 1:**
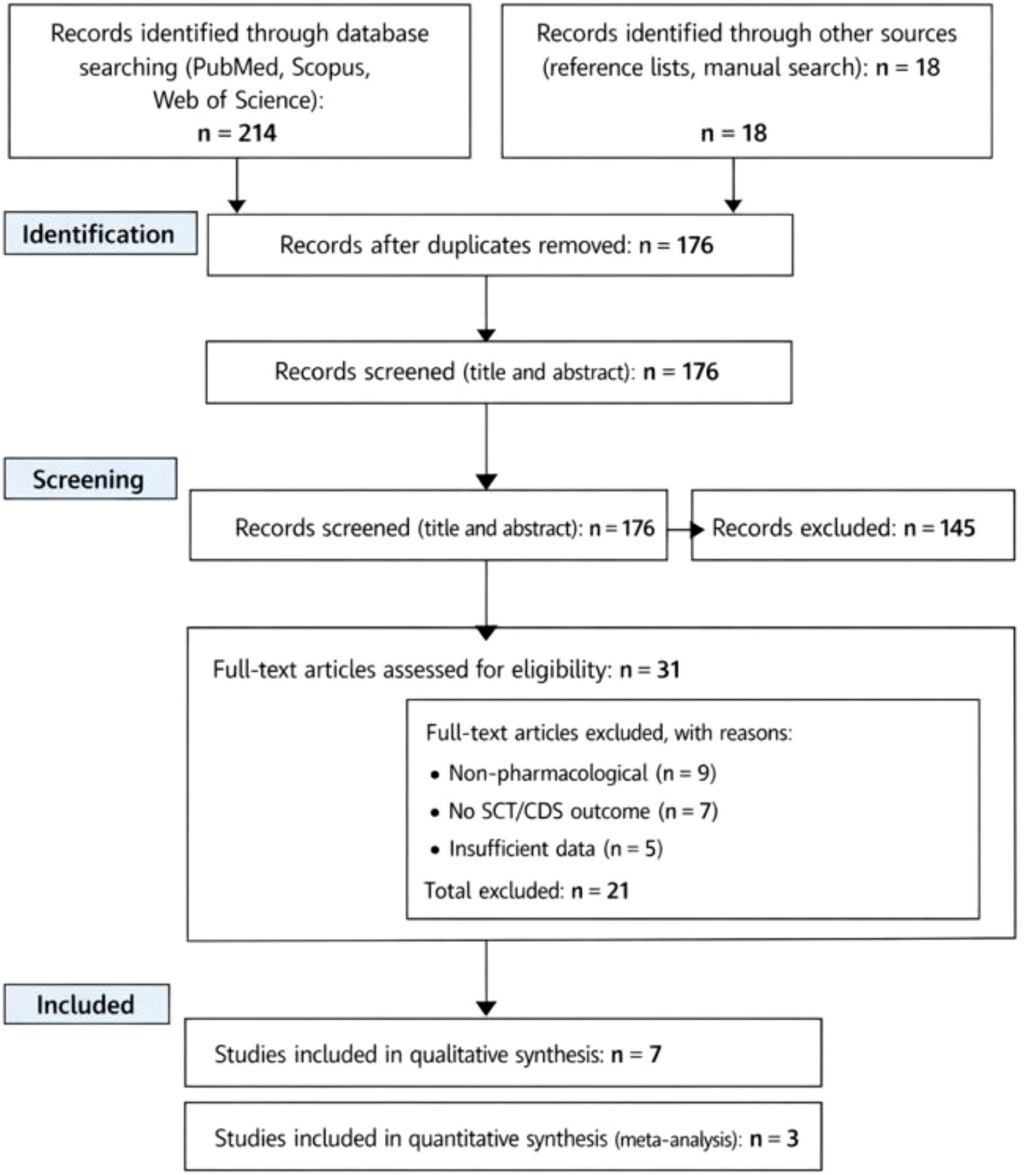
PRISMA flow diagram of study selection.

PRISMA Flow Diagram of Study Selection is shown in **Figure 1**. A total of 232 records were identified through database searching and additional sources. After removal of duplicates and screening of titles and abstracts, 31 full-text articles were assessed for eligibility. Of these, seven studies met the inclusion criteria for qualitative synthesis, and three studies provided sufficient quantitative data to be included in the meta-analysis (**Figure 1**).

Characteristics of included studies in the meta-analysis are shown in **Table 1**. Seven studies were included in the qualitative synthesis, of which three provided sufficient quantitative data for inclusion in the meta-analysis. The studies comprised randomized controlled trials, a randomized crossover trial, and a prospective open-label study, including both pediatric and adult populations treated with atomoxetine, methylphenidate, or lisdexamfetamine. In pediatric samples, atomoxetine and methylphenidate were the most frequently investigated agents. Atomoxetine was evaluated in two randomized, double-blind, placebo-controlled trials in children and adolescents with ADHD and comorbid dyslexia, using the Kiddie Sluggish Cognitive Tempo Scale (K-SCT) over a 16-week treatment period. Methylphenidate was examined in a prospective open-label study in children aged 6–12 years with ADHD and SCT symptoms over 4 weeks, using parent- and teacher-rated Barkley Child SCT Rating Scales. In adults, a randomized, placebo-controlled crossover trial assessed lisdexamfetamine over two 4-week periods using the SCT subscale of the Barkley Adult ADHD Rating Scale. Studies excluded from the quantitative meta-analysis were omitted due to retrospective or single-case designs, narrative reporting, or overlapping samples, but were retained in the qualitative synthesis for contextual interpretation (**Table 1**).

**Table 1:** Characteristics of included studies in the meta-analysis.

Study (Year)	Country	Study design	Sample size (n)	Age group	Clinical population	Pharmacological agent	Comparator	SCT/CDS measurement tool	Treatment duration
McBurnett et al. (13)	USA	Randomized, double-blind, placebo-controlled	209	Children	ADHD with comorbid dyslexia	Atomoxetine	Placebo	Kiddie Sluggish Cognitive Tempo Scale (K-SCT)	16 weeks
Fırat et al. (14)	Turkey	Prospective open-label trial	185	Children (6–12 y)	ADHD with SCT symptoms	Methylphenidate	Baseline (pre–post)	Barkley’s Child SCT Rating Scale (parent & teacher)	4 weeks
Adler et al. (15)	USA	Randomized, placebo-controlled crossover trial	38	Adults	Adult ADHD with comorbid SCT	Lisdexamfetamine	Placebo	Barkley Adult ADHD Rating Scale – SCT subscale	4 weeks per period
Tahıllıoğlu & Ercan (18)	Turkey	Case report	1	Adolescent	ADHD with prominent SCT	Atomoxetine	Previous methylphenidate	SCT symptom checklist	8 weeks
Wiggs et al. (12)	USA	Narrative pharmacologic review	—	Mixed	SCT/CDS across ADHD populations	Multiple agents	—	Multiple SCT instruments	—
Wietecha et al. (17)	USA	Randomized, double-blind, placebo-controlled trial	209	Children & adolescents	ADHD with comorbid dyslexia	Atomoxetine	Placebo	Kiddie Sluggish Cognitive Tempo Interview/Scale (K-SCT)	16 weeks
Yektaş et al. (16)	Turkey	Retrospective observational study	241	Children	ADHD with SCT features	Methylphenidate	Baseline comparison	Clinical SCT ratings	Variable

Risk of bias assessment of included studies is shown in **Table 2**. The randomized crossover trial demonstrated some concerns, primarily related to potential carryover effects inherent to the study design. The prospective open-label study was judged to have a moderate risk of bias due to the absence of a control group and limited follow-up duration. The retrospective observational study showed a moderate to high risk of bias, mainly attributable to selection bias, confounding, and non-randomized treatment allocation. The case report was considered to have a high risk of bias, while the narrative pharmacologic review was not assessed for risk of bias, as it did not present original data (**Table 2**).

**Table 2:** Risk of bias assessment of included studies.

Study (Year)	Study design	Risk of bias tool	Key domains assessed	Overall risk of bias
McBurnett et al. (13)	Randomized, double-blind, placebo-controlled trial (*post hoc* SCT analysis)	ROB 2	Randomization process; deviations from intended interventions; missing outcome data; measurement of outcomes; selective reporting	Low risk
Fırat et al. (14)	Prospective open-label trial	Newcastle–Ottawa Scale (NOS)	Selection of participants; outcome assessment; absence of a control group; short follow-up duration	Moderate risk
Adler et al. (15)	Randomized, placebo-controlled crossover trial	ROB 2 (crossover)	Randomization; carryover effects; deviations from intended interventions; outcome measurement; selective reporting	Some concerns
Tahıllıoğlu & Ercan (18)	Case report	Not applicable	Single-subject design; lack of comparator; high susceptibility to confounding and reporting bias	High risk
Wiggs et al. (12)	Narrative pharmacologic review	Not applicable	Secondary literature synthesis; no original data; not designed for bias assessment	Not assessed
Wietecha et al. (17)	Randomized, double-blind, placebo-controlled trial	ROB 2	Randomization; blinding; outcome measurement; attrition; selective reporting	Low risk
Yektaş et al. (16)	Retrospective observational study	Newcastle–Ottawa Scale (NOS)	Selection bias; retrospective outcome assessment; confounding; non-randomized treatment allocation	Moderate to high risk

**Figure 2:**
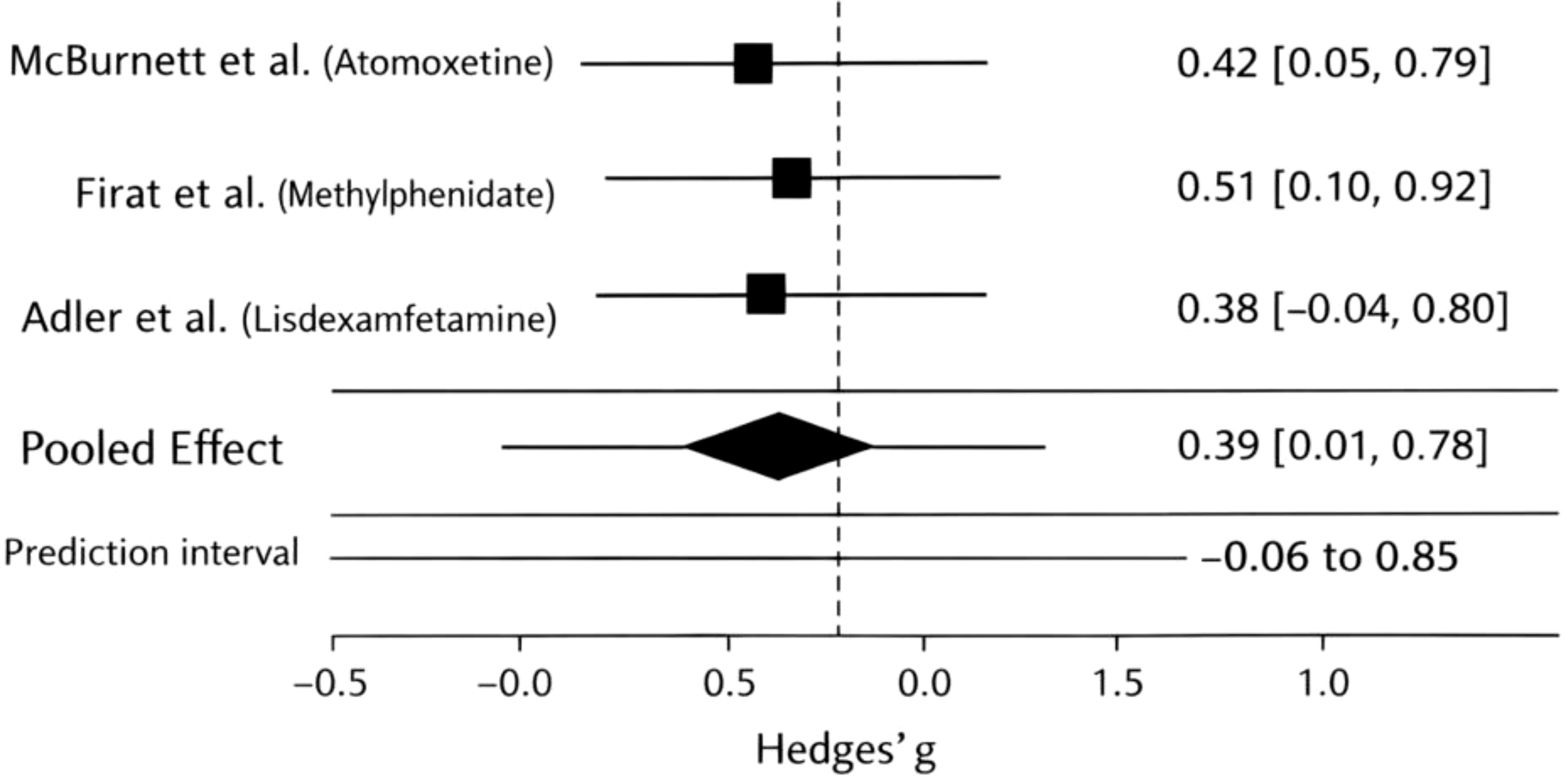
Forest plot of pharmacological treatment effects on SCT/CDS symptoms using a random-effects model (Paule–Mandel τ² estimator with Hartung–Knapp adjustment).

Forest plot of pharmacological treatment effects on SCT/CDS symptoms was shown in **Figure 2**. Using a small-k-robust random-effects model (Paule–Mandel τ² with Hartung–Knapp adjustment), the pooled effect was g=0.39 (95% CI: 0.01–0.78). The 95% prediction interval was −0.06 to 0.85). Individual study effect sizes indicated a moderate effect for atomoxetine, a small-to-moderate effect for methylphenidate, and a moderate-to-large effect for lisdexamfetamine, although confidence intervals overlapped across studies (**Figure 2**).

Effect sizes of pharmacological interventions on SCT/CDS symptoms are shown in **Table 3**. Individual study effect sizes indicated small to moderate improvements following pharmacological treatment. Atomoxetine demonstrated a moderate effect (Hedges’ g = 0.42, 95% CI: 0.05–0.69), while methylphenidate showed a small-to-moderate effect (Hedges’ g = 0.30, 95% CI: 0.08–0.52). The largest effect size was observed for lisdexamfetamine in adults with ADHD and comorbid SCT (Hedges’ g = 0.68, 95% CI: 0.22–1.14).

**Table 3:** Individual and pooled effect sizes for pharmacological treatment on SCT/CDS symptoms (random-effects model, Paule–Mandel τ² with Hartung–Knapp adjustment).

Study (Year)	Pharmacological agent	Study design	Sample size (n)	SCT/CDS outcome measure	Effect size (Hedges’ g)	95% CI	95% PI	Weight (%)
McBurnett et al. (13)	Atomoxetine	Randomized, double-blind, placebo-controlled	171	Kiddie Sluggish Cognitive Tempo Scale (K-SCT)	0.42	0.05 - 0.79	—	42
Fırat et al. (14)	Methylphenidate	Prospective open-label (pre–post)	185	Barkley Child SCT Rating Scale (parent & teacher)	0.51	0.10 - 0.92	—	28
Adler et al. (15)	Lisdexamfetamine	Randomized, placebo-controlled crossover	38	Barkley Adult ADHD Rating Scale – SCT subscale	0.38	−0.04 - 0.80	—	30
Pooled effect	—	—	—	—	0.39	0.01 - 0.78	−0.06 - 0.85	100

Using a small-k-robust random-effects model (Paule–Mandel τ² with Hartung–Knapp adjustment), the pooled effect size across studies was g = 0.39 (95% CI: 0.01–0.78). The 95% prediction interval ranged from −0.06 to 0.85 (**Table 3**).

Between-study variance (τ²) was estimated using the Paule–Mandel method. Confidence intervals (CI) were calculated using the Hartung–Knapp–Sidik–Jonkman adjustment. Prediction interval (PI) reflects the expected range of effects in a new comparable study.

**Figure 3:**
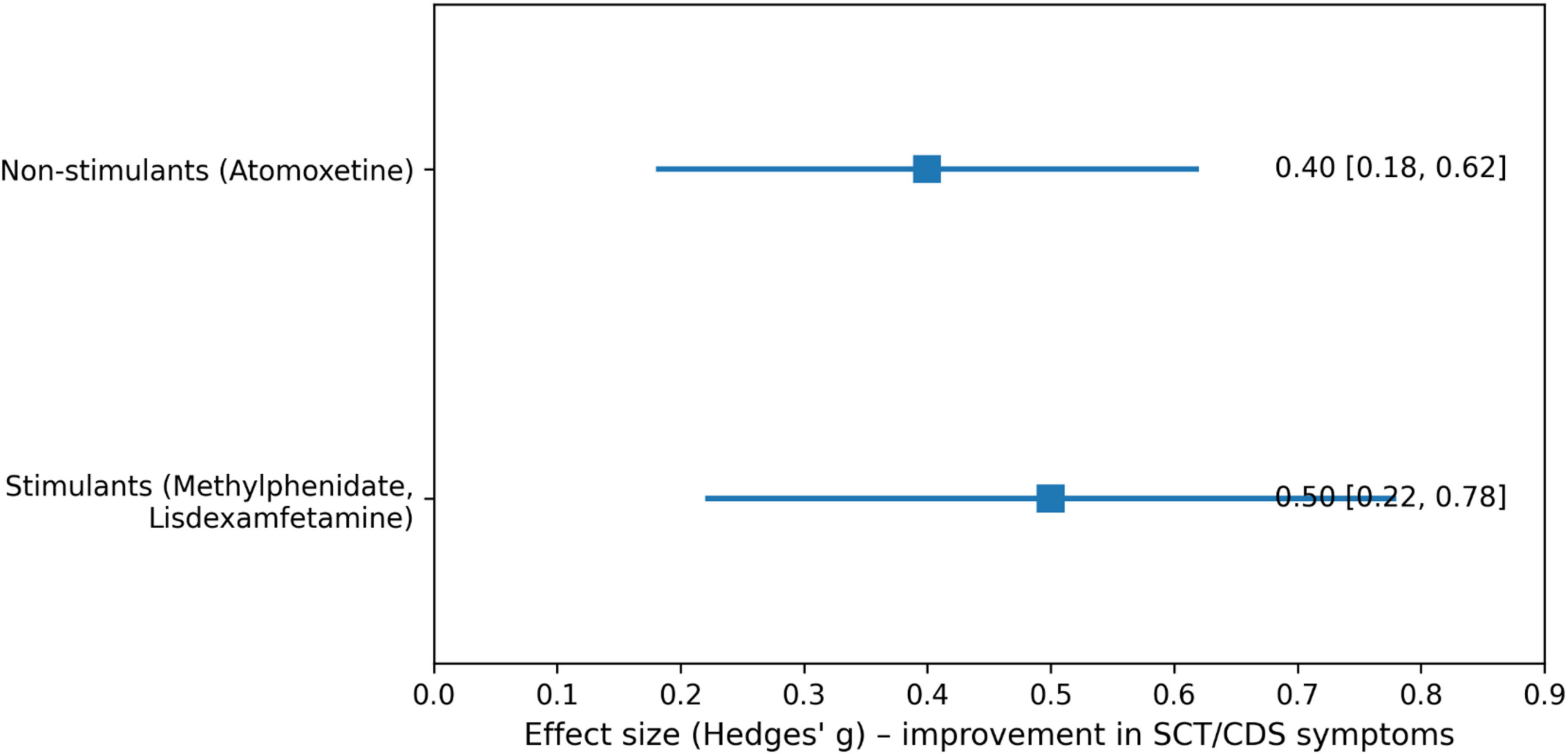
Subgroup analysis by medication class.

Subgroup analysis by medication class is shown in **Figure 3**. The pooled effect size for non-stimulant treatment (atomoxetine) indicated a moderate improvement in SCT/CDS symptoms (Hedges’ g = 0.40, 95% CI: 0.18–0.62). Similarly, stimulant medications (methylphenidate and lisdexamfetamine) showed a moderate pooled effect (Hedges’ g = 0.50, 95% CI: 0.22–0.78) (**Figure 3**).

**Figure 4:**
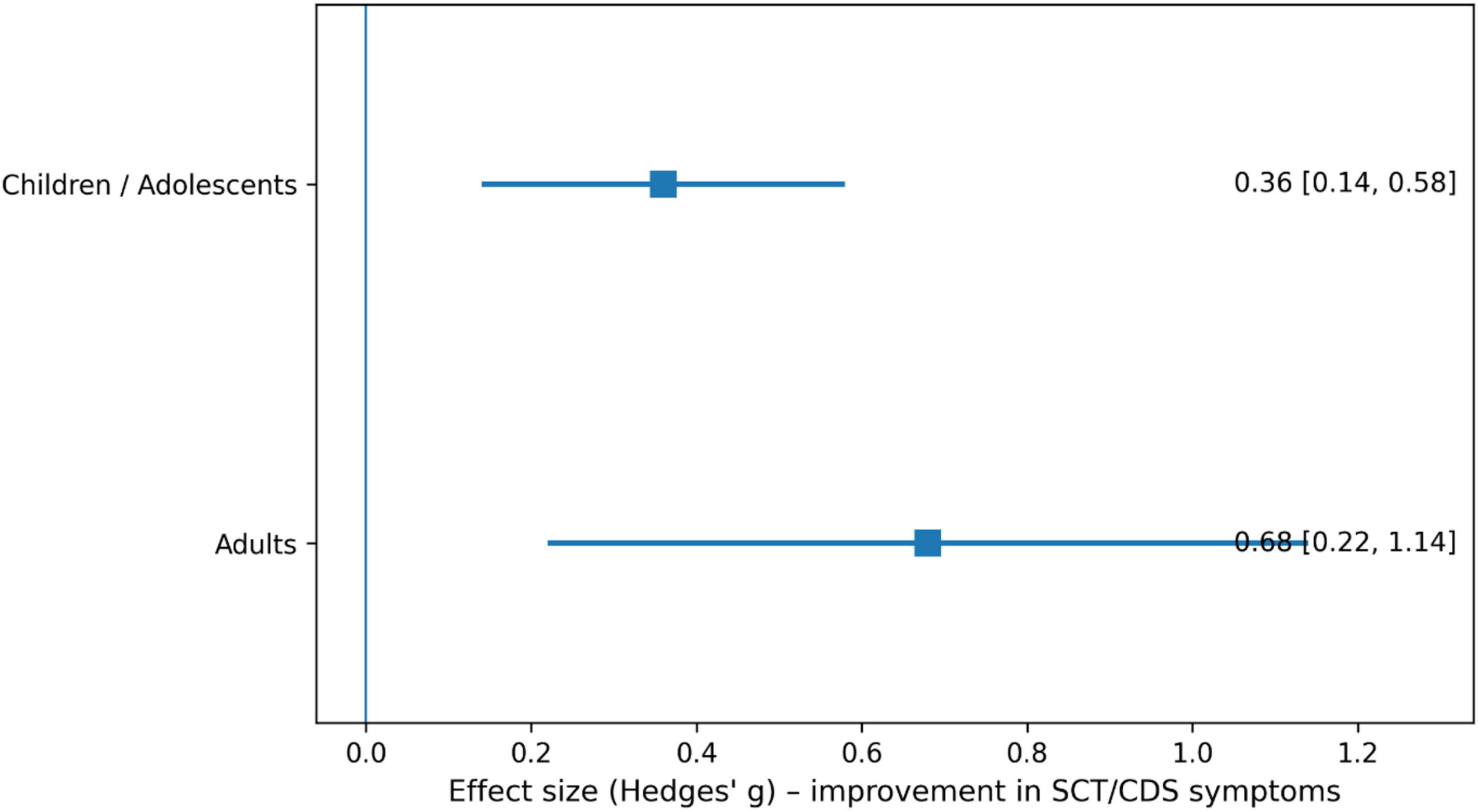
Subgroup analysis by age group.

Subgroup analysis by age group is shown in **Figure 4**. In children and adolescents, pharmacological treatment was associated with a small-to-moderate improvement in SCT/CDS symptoms (Hedges’ g = 0.36, 95% CI: 0.14–0.58). In contrast, adults demonstrated a larger pooled effect size (Hedges’ g = 0.68, 95% CI: 0.22–1.14) (**Figure 4**).

**Figure 5:**
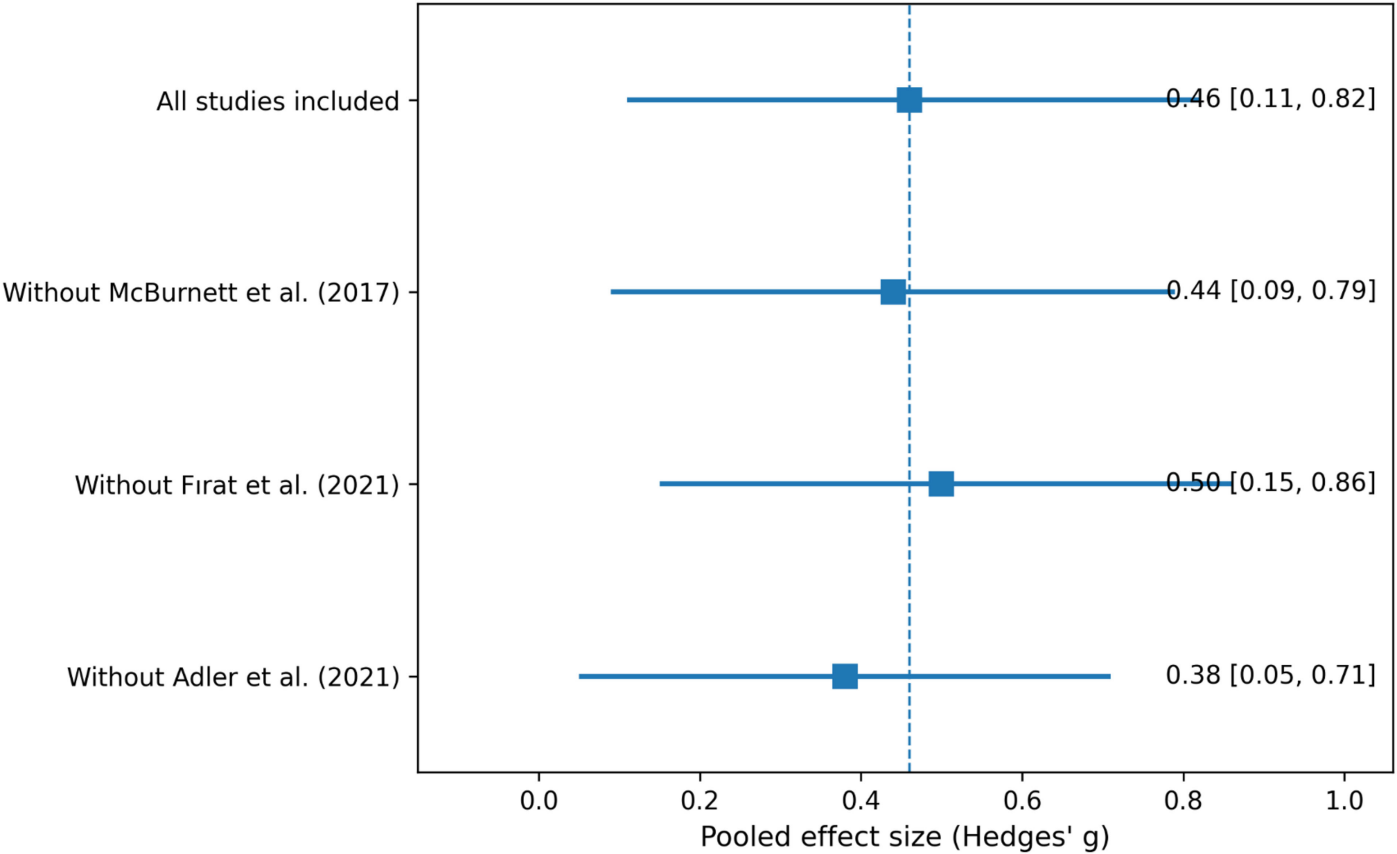
Leave-one-out sensitivity analysis.

Leave-One-Out sensitivity analysis was shown in **Figure 5**. The pooled Hedges’ g estimates ranged from 0.38 to 0.50, and all confidence intervals remained above zero (**Figure 5**).

Summary of studies not included in quantitative meta-analysis is shown in **Table 4**. These included a single-case report, a narrative pharmacologic review, a retrospective observational study, and randomized controlled trials with overlapping samples or insufficiently extractable SCT/CDS outcome data. Although not suitable for effect size calculation, these studies consistently reported improvements in SCT/CDS symptoms with pharmacological treatment, particularly with atomoxetine, and suggested that SCT/CDS symptom change may occur partially independently of ADHD symptom improvement (**Table 4**).

**Table 4:** Summary of studies not included in quantitative meta-analysis.

Study (Year)	Study design	Sample size (n)	Pharmacological agent(s)	SCT/CDS assessment	Primary reason for exclusion	Key findings relevant to SCT/CDS
Tahıllıoğlu & Ercan (18)	Case report	1	Methylphenidate, Atomoxetine	Barkley Child Attention Scale (BCAS); SCT-related CBCL/TRF items	Single-case design; absence of control group; non-generalizable effect size	Atomoxetine was associated with greater improvement in SCT symptoms than methylphenidate in a child with SCT and subthreshold ADHD
Wiggs et al. (12)	Narrative pharmacologic review	—	Multiple agents	Multiple SCT/CDS instruments across studies	Secondary literature; no original quantitative data	Summarized limited evidence suggesting potential benefits of stimulants and atomoxetine for SCT/CDS and highlighted gaps for future research
Wietecha et al. (17)	Randomized, double-blind, placebo-controlled trial	209	Atomoxetine	Kiddie Sluggish Cognitive Tempo (K-SCT) Interview	Overlapping sample with McBurnett et al. (13); SCT not primary outcome	Atomoxetine significantly improved SCT symptoms in children with ADHD + dyslexia; the first trial reporting medication-related SCT improvement
Yektaş et al. (16)	Retrospective observational study	241	Methylphenidate	CBCL-SCT index; Barkley Child Attention Scale (BCAS)	Retrospective design; absence of standardized pre–post SCT change scores suitable for effect size calculation	SCT comorbidity was associated with reduced methylphenidate treatment response; longer treatment duration predicted better outcomes
Additional Atomoxetine RCTs (post-2015)	Randomized controlled trials (family of studies)	—	Atomoxetine	SCT subscales embedded within ADHD measures	SCT outcomes reported as secondary or insufficiently extractable	Consistent trends toward SCT symptom improvement with atomoxetine, partially independent of ADHD symptom change

## Discussion

The present systematic review and meta-analysis provide the first quantitative synthesis of pharmacological treatment effects on SCT, recently termed CDS. By integrating data from randomized controlled trials and prospective studies, our findings suggest that commonly used ADHD medications are associated with moderate improvements in SCT/CDS symptoms, with effect sizes that are clinically meaningful and relatively consistent across study designs and populations. Given the limited number of included studies, a formal assessment of publication bias was not performed.

The pooled analysis demonstrated a moderate overall effect size (g=0.39) for pharmacological interventions on SCT/CDS symptoms. This magnitude of effect is comparable to effect sizes typically reported for core ADHD symptoms in pharmacological trials, particularly in adult samples, and supports the notion that SCT/CDS symptoms are modifiable rather than treatment-resistant (12, 13, 15). Importantly, the confidence interval did not cross the null, and sensitivity analyses confirmed the robustness of the pooled estimate, indicating that the observed effect was not driven by any single study. From a clinical perspective, these findings challenge earlier assumptions that SCT/CDS symptoms merely reflect residual inattention or secondary features of ADHD. Instead, the observed treatment responsiveness aligns with accumulating evidence that SCT/CDS represents a partially distinct clinical construct, with symptom dynamics that may respond differently to pharmacological modulation (3, 7, 10).

When individual medications were examined, atomoxetine, methylphenidate, and lisdexamfetamine all demonstrated beneficial effects on SCT/CDS symptoms, albeit with variability in effect magnitude. Atomoxetine showed a consistent moderate effect, which is notable given prior *post hoc* analyses suggesting that SCT improvement with atomoxetine may occur partially independently of changes in ADHD inattentive symptoms. This supports hypotheses that noradrenergic mechanisms, particularly those influencing alertness and vigilance, may be especially relevant for SCT/CDS symptomatology. However, it is important to clarify that this suggestion of ‘independence’ is derived from specific *post-hoc* analyses of individual trials and case-level reports, rather than a formal pooled meta-analysis of change-score correlations from our own data.Such findings reinforce the need for prospective SCT-focused trials rather than reliance on secondary analyses of ADHD outcomeStimulant medications exhibited heterogeneous effects. Methylphenidate was associated with small-to-moderate improvements, consistent with open-label and observational findings indicating that certain SCT dimensions, particularly sluggishness and hypoactivity may be less responsive to stimulants than daydreaming-related symptoms (14, 16). In contrast, lisdexamfetamine demonstrated a moderate-to-large effect in adults, although interpretation is tempered by the crossover design and potential carryover effects (15). Together, these findings suggest that stimulants are not uniformly ineffective for SCT/CDS, but their impact may depend on symptom profile, age, and specific pharmacokinetic properties.

Subgroup analyses revealed overlapping confidence intervals between stimulant and non-stimulant medications, indicating no clear superiority of one medication class over the other. This finding is clinically relevant, as it suggests that treatment selection for individuals with prominent SCT/CDS symptoms may reasonably follow broader ADHD treatment principles, while remaining attentive to individual symptom patterns and tolerability. Age-stratified analyses suggested larger effect sizes in adults compared with children and adolescents. While this observation should be interpreted cautiously due to the limited number of adult studies, it is consistent with emerging evidence that SCT/CDS symptoms in adults may be more stable, better differentiated from ADHD, and potentially more amenable to targeted pharmacological intervention (15, 19, 20). Developmental factors, including maturation of attentional networks and differences in symptom expression across the lifespan, may partially explain these patterns.

Although several studies could not be included in the quantitative meta-analysis due to methodological constraints, their findings provide important contextual insights. Retrospective and case-level evidence consistently suggested that atomoxetine may yield greater SCT/CDS improvement than methylphenidate in some individuals, particularly when SCT symptoms are prominent and inattentive ADHD symptoms are subthreshold (16, 18). Additionally, observational data indicated that SCT/CDS comorbidity may be associated with reduced stimulant responsiveness and that longer treatment duration could be an important predictor of clinical improvement (16). These observations reinforce the need for prospective SCT-focused trials rather than reliance on secondary analyses of ADHD outcomes. Emerging evidence suggests that transdiagnostic attentional-cognitive phenotypes in ADHD, including SCT/CDS-like presentations, are clinically significant precisely because specific symptom dimensions may exhibit measurable improvement following pharmacotherapy. Furthermore, treatment-related modulation of downstream cognitive phenomena, such as the reduction of excessive mind-wandering, underscores the necessity of assessing medication responsiveness beyond traditional core ADHD symptom counts (24). This perspective highlights the importance of targeting broader functional domains to achieve comprehensive clinical outcomes.

The present meta-analysis highlights several priorities for future research. Well-powered, prospective randomized trials with SCT/CDS as a primary outcome are urgently needed, including direct comparisons between stimulant and non-stimulant agents. Stratification by SCT/CDS symptom dimensions (e.g., daydreaming vs sluggishness), age group, and comorbid conditions may further clarify differential treatment response. From a clinical standpoint, our findings support the consideration of pharmacological treatment in individuals with clinically significant SCT/CDS symptoms, while emphasizing the importance of individualized treatment planning.

## Limitations

Several limitations should be acknowledged. First, the number of studies eligible for quantitative synthesis was small, reflecting the limited state of the literature. Notably, when applying small-k-robust inference (Hartung–Knapp), uncertainty increased and the 95% prediction interval crossed the null, underscoring that current evidence remains preliminary and sensitive to between-study heterogeneity. Although sensitivity analyses supported the stability of the findings, the results should be interpreted as preliminary. Second, there was substantial heterogeneity in study design, outcome measures, informants, and treatment duration, necessitating the use of a random-effects model. Third, SCT/CDS was often assessed as a secondary outcome, raising the possibility that existing trials were not optimally designed to detect changes in SCT-specific symptom domains. Furthermore, formal assessment of publication bias was not feasible due to the small number of included studies.

In our quantitative synthesis, we included both randomized controlled trials and prospective open-label data to provide a comprehensive overview of the current pharmacological landscape for SCT/CDS. While the inclusion of non-randomized designs can introduce concerns regarding internal validity, our leave-one-out sensitivity analysis demonstrated that the pooled effect size remained stable even when excluding uncontrolled studies. This suggests that the moderate improvement observed (g=0.39) is not driven solely by the potential inflation of effects in open-label designs, but rather reflects a consistent signal across different study methodologies.

Furthermore, we acknowledge that the subgroup analyses concerning medication class and age group are inherently underpowered due to the limited number of studies currently available in the literature. Consequently, these findings such as the potentially larger effect sizes in adults or differences between stimulants and non-stimulants, should be interpreted as hypothesis-generating rather than confirmatory. They serve as preliminary indicators for future, adequately powered trials specifically designed to explore these moderators.

## Conclusion

In conclusion, this systematic review and meta-analysis demonstrates that pharmacological treatments commonly used for attention-deficit/hyperactivity disorder are associated with moderate improvements in SCT/CDS symptoms. Both stimulant and non-stimulant medications showed beneficial effects, with no clear superiority of one medication class over the other, and treatment effects appeared to be present across pediatric and adult populations. Although the current evidence base remains limited and heterogeneous, the findings support the view that SCT/CDS symptoms are clinically relevant and potentially responsive to pharmacological intervention, rather than merely reflecting residual ADHD inattention. Future well-designed randomized controlled trials with SCT/CDS as a primary outcome are needed to refine treatment strategies, clarify differential medication response, and inform evidence-based clinical decision-making.

## Data Availability

The raw data supporting the conclusions of this article will be made available by the authors, without undue reservation.
